# Evaluation of the SeedCounter, A Mobile Application for Grain Phenotyping

**DOI:** 10.3389/fpls.2016.01990

**Published:** 2017-01-04

**Authors:** Evgenii Komyshev, Mikhail Genaev, Dmitry Afonnikov

**Affiliations:** ^1^Laboratory of Evolutionary Bioinformatics and Theoretical Genetics, Department of Systems Biology, Institute of Cytology and Genetics Siberian Branch of Russian Academy of Sciences (SB RAS)Novosibirsk, Russia; ^2^Chair of Informational Biology, Novosibirsk State UniversityNovosibirsk, Russia

**Keywords:** wheat grain, phenotyping, computer image analysis, mobile devices, Android

## Abstract

Grain morphometry in cereals is an important step in selecting new high-yielding plants. Manual assessment of parameters such as the number of grains per ear and grain size is laborious. One solution to this problem is image-based analysis that can be performed using a desktop PC. Furthermore, the effectiveness of analysis performed in the field can be improved through the use of mobile devices. In this paper, we propose a method for the automated evaluation of phenotypic parameters of grains using mobile devices running the Android operational system. The experimental results show that this approach is efficient and sufficiently accurate for the large-scale analysis of phenotypic characteristics in wheat grains. Evaluation of our application under six different lighting conditions and three mobile devices demonstrated that the lighting of the paper has significant influence on the accuracy of our method, unlike the smartphone type.

## Introduction

The grains per ear and grain size are important characteristics of cereal yield. Seed counting and morphometry “by eye” is laborious. Therefore, various approaches have been suggested for efficient grain morphometry using image processing techniques (Granitto et al., 2005; Pourreza et al., 2012; Tanabata et al., 2012). Most of these approaches were implemented using desktop PC software for analyzing grain images on a light background obtained using either a digital camera or a scanner (Herridge et al., 2011; Tanabata et al., 2012; Whan et al., 2014). These approaches allow users to estimate a large number of grain morphometric parameters describing shape and color (Bai et al., 2013). They also facilitate methods for identifying the cereal variety using grain images (Wiesnerová and Wiesner, 2008; Chen et al., 2010; Zapotoczny, 2011), determining seed moisture content and predicting semolina yield in durum wheat (Novaro et al., 2001; Tahir et al., 2007). Duan et al. (2011) developed a labor-free engineering solution for high throughput automatic analysis of rice yield-related traits including the number of total spikelets, the number of filled spikelets, the 1000-grain weight, the grain length, and the grain width. Roussel et al. (2016) proposed a detailed analysis of seed shape and size. They used 3D surface reconstruction from the silhouettes of several images obtained by rotation of a seed in front of a digital camera. This method was implemented further in the *pheno*Seeder robotic platform (Jahnke et al., 2016), which was designed for the high-quality measurement of basic seed biometric traits and mass from which seed density is calculated. Strange et al. (2015) used X-ray computed tomography for the *in situ* determination of grain shape. The engineering facilities for grain morphometry demonstrate high performance and precision; however, they are installed in a limited number of plant research laboratories. There is still a need for low cost, high-throughput methods of grain analysis (Whan et al., 2014).

Large-scale breeding experiments require processing substantial phenotypic data, often in field conditions and thus without access to desktop computers and scanners. In this case, a digital camera is a viable option, but the images must be subsequently copied to a laptop or PC.

Modern mobile devices (smartphones and Internet tablets) contain digital cameras with high resolution. Mobile devices have multicore processors with sufficient computational power for image processing and analysis. These features allow users to capture and process images wherever necessary. A number of applications for mobile devices have been developed for the morphometry of plant organs. Leafsnap (Kumar et al., 2012) is able to identify plant species in real time based on their leaf images: a user takes pictures of a plant leaf using a mobile device and sends the images from the camera to a remote server where they are processed. Leaf Doctor (Pethybridge and Nelson, 2015) is another mobile application that estimates the percentage of disease severity based on leaf images in a semi-automated manner. Mobile devices can also serve as efficient tools to estimate soil-color (Gómez-Robledo et al., 2013).

In this work, we present a mobile application, SeedCounter, for the Android platform that performs automated calculation of morphological parameters of wheat grains using mobile devices in field conditions (without computer facilities). The application estimates the number of grains scattered on a sheet of A4, Letter, Legal, A3, A4, A5, B4, B5, or B6 paper and morphological parameters such as length, width, area, and distance between the geometric center of mass of the grain and the point of intersection of its principal axes.

We conducted several seed counting tests under controlled lighting conditions and daylight to estimate software performance. We demonstrated that the SeedCounter can estimate the number of grains in an image and their size with high accuracy, but performance is dependent on lighting conditions.

## Materials and Methods

### Getting Images

The program input is a color image of grains placed arbitrarily on a sheet of white paper (A4, Letter, Legal, A3, A5, B4, B5, or B6). We recommend minimizing any contact between grains. To reduce errors, users should provide the following conditions for image capture: the paper sheet should be placed on a dark background and bright side lighting should be avoided.

**FIGURE 1:**
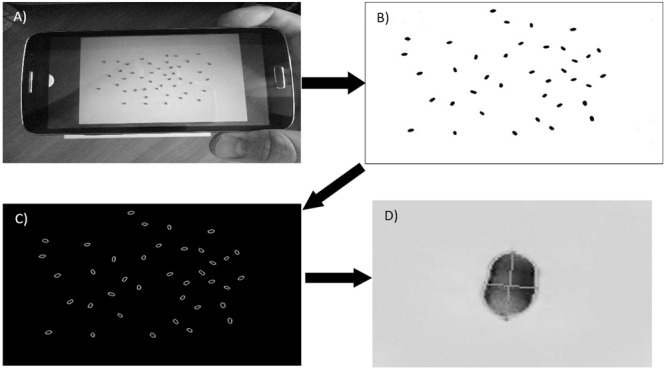
**The main steps of grain recognition on a sheet of paper. (A)** Capturing an image using the camera of the mobile device and paper recognition. **(B)** The image after affine transformation and binarization. **(C)** Grain contours identified on the image. **(D)** Grain image with the major axes shown by crossed lines.

The boundaries of the paper sheet on the background should be parallel to the sides of the frame (**Figure 1A**). The fixed size of the paper makes it possible to calculate the scale of the image and evaluate the grain sizes in metric units. The SeedCounter application receives images directly from the camera of the mobile device.

### Image Processing Algorithm

The algorithm is implemented using the OpenCV image processing library (Howse, 2013; Dawson-Howe, 2014) and consists of several steps.

### Paper Sheet Recognition

The paper sheet is recognized as a light area of tetragonal shape on a dark background. For recognition, the original color image (**Figure 1**) is converted to grayscale by the cvtColor() function. To determine the area of the sheet, an adaptive binarization of the entire image is performed by the adaptiveThreshold() function, and the canny() function is used for paper boundary detection. The set of lines close to the sheet boundaries is generated by the houghLinesP() function with the length parameter varying from 20 to 80% of the respective image side. Due to distortions on the image, not all of these lines for the same side are parallel and lines at the adjacent sides are not perpendicular. Therefore, to select lines approximating the paper boundaries, we cluster them with respect to their mutual angle and distance, yielding four clusters of lines corresponding to the paper sides. For each cluster, we reconstruct a sheet boundary line with the smallest distance from the pixels of the cluster lines. The intersections between the sheet boundary lines determine the vertices of the paper tetragonal image. If the paper shape on the image deviates from rectangular, affine transformations convert it to rectangular. This step is performed using the getPerspectiveTransform() function for transformation matrix calculations, and the warpPerspective() function is used to transform the image, making the opposite edges parallel and all angles equal to 90°.

### Grain Identification and Morphometry

Grains are identified as contours by applying the findContours() function to the image fragment corresponding to the paper sheet. We make a further adjustment of the grain boundaries using local Hue Saturation Value (HSV) channel binarization for the neighboring regions of the original image. Local binarization reduces the influence of shadowing during grain boundary determination. It includes converting a local image segment to HSV color space and a subsequent conversion into grayscale based on calibration parameters and color histograms. The resulting channel reflects the degree of conformity of image pixels to the grain color. The local binarization yields more accurate determinations of grain boundaries.

The marked watershed method (Roerdink and Meijster, 2000), as implemented in the watershed() function, is used to resolve the boundaries of seed grains that are in contact with one another. The resulting contours are approximated by grain ellipsoids, allowing for estimates of the size of the major and minor principal axes corresponding to the length and the width of the grain (**Figure 1D**). SeedCounter additionally identifies the grain image area and the distance between the geometric center of mass and the point of intersection of the principal axes.

### Android Application Interface

**FIGURE 2:**
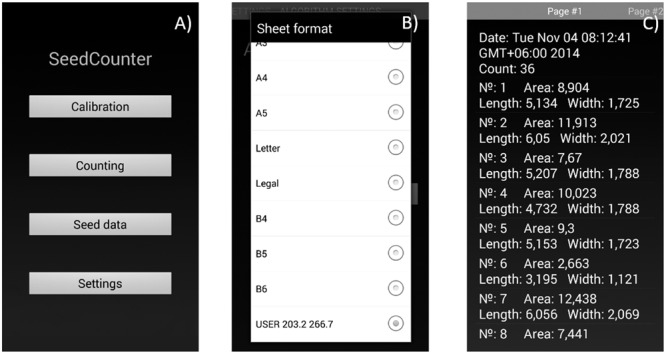
**The SeedCounter application interface. (A)** Main menu. **(B)** Selection of the paper size. **(C)** Output screen indicating the results of measurements (grain count and length/width/area for each grain).

The mobile application user can adjust image processing and seed recognition parameters by using the ‘Calibration’ option on the main menu (**Figure 2A**). The user should provide a single seed on the paper, process the image and verify that the algorithm identifies the seed correctly and marks it as a red polygon. The algorithm parameters at this stage are saved automatically. The user can also use the program menu (**Figure 2B**) to define the size of the paper sheet (including user-defined sizes) and the camera and image resolutions to enable the touching seed separation algorithm and HSV binarization.

Data on the number of counted seeds and seed shape parameters for each seed are stored in XML format and can be displayed using the ‘Seed data’ menu (**Figure 2C**). The user can view the data, delete it, export in tsv format or send it to the SeedCounter web-server. In the last case, the user obtains the data URL that allows the uploading of the data in the web-browser.

### Accuracy Estimation

We considered two types of errors. First, we estimated the accuracy of the grain number identification. Fifty wheat grains of the same variety were poured onto a sheet, and the number of grains was estimated by SeedCounter. After that, one grain was removed from the sheet, the grains were shuﬄed (no control for the grain separation), and the number of grains was estimated again. This procedure was repeated 40 times. We performed this measurement series using different mobile devices, camera resolutions, and illumination conditions. For each series of grain number estimations, we calculated the mean absolute error (MAE) and the mean absolute percentage error (MAPE) as follows:

$$\begin{array}{c} MAE=\frac{1}{M}\sum_{j=1}^{j=M}|N_{j}{-N}_{j}^{{}^{\prime }}|\\ MAPE=\frac{100\%}{M}\sum_{j=1}^{j=M}\left(\frac{\left|N_{j}{-N}_{j}^{{}^{\prime }}\right|}{N_{j}}\right) \end{array}$$

where *j* is the image number in the experiment, *N*_j_ is the number of grains on the sheet, *N*_j_′ is the number of grains estimated by SeedCounter and *M* = 40 is the number of images in the experiment. The error in seed grain number estimation increases as the *MAE* [Eq. (1)] and *MAPE* [Eq. (2)] values increase. If the *MAE* and *MAPE* values are close to 0, the error is low. We additionally estimated the Pearson correlation coefficient, *r*_N_, between *N*_j_ and *N*_j_′. The closer *r_N_* is to unity, the smaller the error in the grain number estimates.

Second, we evaluated the accuracy of the grain length and width estimation. We measured the length and width of 250 grains of five wheat varieties, with each grain placed in a strict order, using a Carl Zeiss Aioscop 2 plus microscope equipped with a digital camera with the AxoCamHRc TV2/3c 0.63 adapter. We placed grains on the paper sheet in the same order and applied the SeedCounter software to estimate their length and width. A series of morphometric measurements of the 250 grains was performed using different mobile devices, camera resolutions and illumination conditions. For each experiment, we calculated MAE [Eq. (1))] separately for length and width and calculated the average values. The same procedure was used to calculate MAPE [Eq. (2)] for the width and length. The Pearson correlation coefficients, *r*_w_ and *r*_l_, were calculated for these parameters separately.

To compare the accuracy of SeedCounter applications with available software, we compared our results with measurements obtained using the application SmartGrain (Tanabata et al., 2012) running on a personal computer (Intel Core i7, 2400 MHz, 4 Gb RAM) and images from the scanner HP Scanjet 3800 with 600 dpi.

### Experimental Conditions

We evaluated the accuracy of the morphometric parameter estimation of grains using the following three mobile devices running Android OS at maximal camera resolution: the smartphones Samsung Galaxy Grand 2, Sony Ericsson XPERIA pro mini, and the Internet tablet DNS AirTab m101w. Characteristics for these devices are presented in Supplementary Table S1.

We used the following three types of lighting devices: A 11-W daylight lamp (color temperature 4000 K, luminous flux 900 lm), a 5-W daylight lamp (4000 K, 400 lm), and a 35-W halogen lamp (2700 K, 190 lm). Four types of artificial lighting were used, as follows: a 11-W daylight lamp (L1); a 11-W daylight lamp and two 5-W daylight lamps (L2); a 11-W daylight lamp and four 5-W daylight lamps (L3); and a 11-W daylight lamp, four 5-W daylight lamps, and a halogen lamp (L4). The lamps were set at a height of 60 cm above the sheet of paper. The sheet was placed on a table with a dark top, and the experiments were performed in a dark room. To assess the accuracy of the measurements in the daylight, we also measured the grains without using artificial lighting in cloudy weather indoors and on a clear day outdoors. Details of the experimental conditions are listed in **Table 1**.

**Table 1:** Light conditions for measuring the accuracy of the wheat grain morphometry.

Number	Lighting facilities	Luminous flux (lux)	Light temperature
L1	11-W daylight lamp	900 lm	4000K
L2	11-W daylight lamp, 2 × 5-W daylight lamps	1700 lm	4000K
L3	11-W daylight lamp, 4 × 5-W daylight lamps	2500 lm	4000K
L4	11-W daylight lamp, 4 × 5-W daylight lamps, 35-W halogen lamp	2690 lm	4000 and 2700K
L5	Daylight, cloudy day, indoors	(1280 lux)	–
L6	Daylight, sunny day, outdoors	(656000 lux)	–

*Lux units were used to evaluate the light intensity of natural lighting conditions.*

We used two-way ANOVA tests to estimate the influence of device type and lighting conditions on grain number and shape accuracy. We considered device type and lighting to be independent variables and error estimates (MAE and MAPE) to be dependent variables. The Statistica 6.0 software was used to perform this test.

### Wheat Varieties

We used the grains from the following five wheat varieties from the cereal collection of the Chromosome engineering laboratory, Institute of Cytology and Genetics SB RAS: Alen’kaya 1102 II-12, 84/98w 99 II-13, Synthetic 6x x-12, Purple Chance 4480 II-03, and Alcedo n-99. Plants were grown in a field near Novosibirsk in 2014. These varieties have grains with different shapes and sizes. The variety Alcedo is oval in shape and has an average length of ∼7 mm and width of ∼3.6 mm. The Synthetic variety has an elongated grain shape and an average length of ∼8 mm and width of ∼2.3 mm. The Alen’kaya variety has smaller dimensions, with an average length of ∼5 mm and an average width of ∼2.4 mm. The 84/98w and Purple Chance varieties are similar in appearance and have an average length/width of 6.5/2.6 mm and 7/2.9 mm, respectively.

## Results

The SeedCounter mobile application for Android devices is free to download at the Android Play Store ^1^). The SeedCounter application requires a minimum of Android API version 15, and Oracle/Sun JDK 6 or 7 is recommended. SeedCounter uses the OpenCV library for image processing. SeedCounter is distributed under the BSD (Berkley Software Distribution) license.

The grain number estimation accuracies for different experiment series are shown in **Table 2**. The table shows that the MAE [Eq. (1)] of the estimate of the number of grains on the sheet is close to 1% and that the MAPE [Eq. (2)] is close to 2%. A more detailed analysis showed that the largest errors in counting the number of grains occur if two or more grains on the paper are in contact and that under poor lighting conditions, the algorithm does not separate most of the grains. If the grains on the sheet are all separated, the seed counting error vanishes.

**Table 2:** Evaluation of the accuracy of wheat grain counting using the SeedCounter mobile application.

Experiment ID	MAE^a^ (mm)	MAPE^a^ (%)	*r* (*N*_j_, *N*_j_′)^a^
Sam_L1	1.425	0.035	0.996
Sam_L2	1.375	0.036	0.994
Sam_L3	0.65	0.015	0.998
Sam_L4	0.975	0.024	0.997
Sam_L5	1.15	0.029	0.992
Sam_L6	0.55	0.017	0.998
Sony_L1	1	0.024	0.995
Sony_L2	0.8	0.019	0.995
Sony_L3	0.675	0.017	0.996
Sony_L4	0.775	0.020	0.997
Sony_L5	0.75	0.018	0.996
Sony_L6	0.775	0.018	0.996
DNS_L1	1.2	0.031	0.997
DNS_L2	0.5	0.012	0.997
DNS_L3	0.125	0.003	0.999
DNS_L4	0.725	0.017	0.998
DNS_L5	1.175	0.030	0.996
DNS_L6	0.775	0.020	0.997

*^a^Mean absolute error (MAE), mean absolute percentage error (MAPE), and Pearson correlation coefficient *r* (*N*_j_, *N*_j_′) between the actual number and estimated number of seeds.*

The accuracy of length and width estimation for the grains by different devices in different conditions is shown in **Table 3**. The table demonstrates that the grain size estimation accuracy was approximately 0.30 mm (average for all series: 0.31 mm) that is approximately 8% of the linear dimensions of the grain (average for all series: 8.03%). The correlation coefficients between the control length and its estimate in all experiments were not lower than 0.79. For the grain width, this parameter was smaller but greater than 0.67. Both correlation coefficients were significant at *p* < 0.01. Interestingly, errors for grain length estimates for SeedCounter and SmartGrain are close to each other; however, for grain width SmartGrain demonstrates better performance.

**Table 3:** The accuracy of estimates of the length and width of wheat grains by SeedCounter mobile application and SmartGrain.

Experiment ID	MAE^a^ (mm)	MAPE^a^ (%)	*r*_l_^a^	*r*_w_^a^
Sam_L1	0.284	7.453	0.936	0.816
Sam_L2	0.296	7.576	0.928	0.824
Sam_L3	0.283	7.339	0.932	0.822
Sam_L4	0.327	8.306	0.923	0.811
Sam_L5	0.398	9.081	0.797	0.770
Sam_L6	0.349	8.437	0.875	0.769
Sony_L1	0.313	8.277	0.933	0.765
Sony_L2	0.310	8.121	0.931	0.767
Sony_L3	0.298	7.787	0.937	0.777
Sony_L4	0.327	8.418	0.920	0.755
Sony_L5	0.301	7.727	0.913	0.749
Sony_L6	0.338	8.546	0.899	0.730
DNS_L1	0.295	7.852	0.943	0.774
DNS_L2	0.296	7.688	0.935	0.777
DNS_L3	0.287	7.730	0.950	0.779
DNS_L4	0.311	8.229	0.940	0.780
DNS_L5	0.351	8.798	0.890	0.672
DNS_L6	0.346	8.264	0.890	0.787
SmartGrain	0.305	6.973	0.948	0.886

*^a^Mean absolute error (MAE), mean absolute percentage error (MAPE) averaged over length and width, and Pearson correlation coefficients for length (*r*_l_) and width (*r*_w_) between actual and estimated values.*

Average values for different devices under the same conditions are shown in Supplementary Table S2. The mobile devices on average demonstrate the best performance in grain size estimation at L3 lighting conditions (two daylight lamps, luminous flux is 2500 lm). The worst performance was obtained at L5 conditions (cloudy day, indoors).

The two-way ANOVA test showed that the lighting conditions significantly influence the estimation of the grain number and the grain length and width (ANOVA *p*-value < 0.05; **Table 4**). Interestingly, the largest mean MAE [Eq. (1)[for grain counting, 0.458, was obtained for the lighting condition with the lowest luminous flux (L1, 11-W lamp only), whereas the other lighting conditions had lower MAE values: 0.058 for L2, 0.1 for L3, 0.058 for L4, and 0.275 for L5. It should be noted that the seed counting error under conditions without artificial light is smaller than that for the lowest luminous flux but larger than that obtained under all other controlled light conditions. The results shown in **Table 4** demonstrate that device type does not have a significant effect on the grain number/dimension measurements.

**Table 4:** Significance of the influence of the mobile device type and lighting on errors in estimating grain number and dimensions.

Error type	Lighting conditions	Device type
Grain counting, MAE	**0.004**	0.365
Grain counting, MAPE	**0.003**	0.306
Grain dimensions, MAE	**0.036**	0.771
Grain dimensions, MAPE	0.094	0.890

*ANOVA *p*-values of two factors are represented. Bold values are significant (*p* < 0.05)*

**FIGURE 3:**
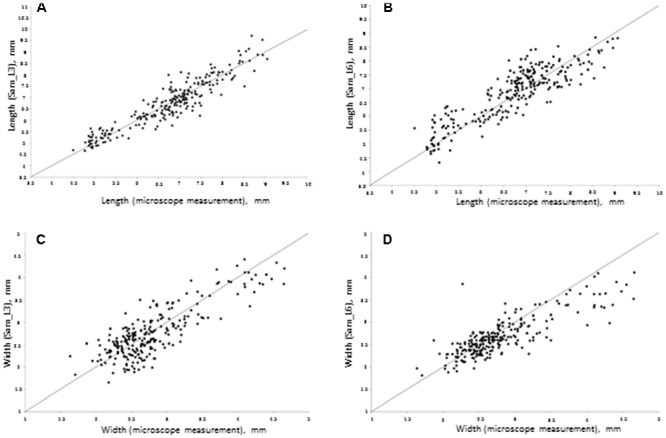
**Scatter plot of seed sizes measured by Samsung mobile device (*Y*-axis) relative to the sizes measured under a microscope (*X*-axis). (A)** Seed length at L3 conditions; regression parameters: intercept = 0.25 (Lower 95%: -0.07, Upper: 95%: 0.57), slope = 0.96 (Lower 95%: 0.91, Upper 95%: 1.01). **(B)** Seed length in L6 conditions; regression parameters: intercept = 1.26 (Lower 95%: 0.86, Upper 95%: 1.64), slope = 0.83 (Lower 95%: 0.77, Upper 95%: 0.88). **(C)** Seed width in L3 conditions; regression parameters: intercept = 0.54 (Lower 95%: 0.33, Upper 95%: 0.73), slope = 0.79 (Lower 95%: 0.73, Upper 95%: 0.87). **(D)** Seed width in L6 conditions; regression parameters: intercept = 0.90 (Lower 95%: 0.58, Upper 95%: 0.68), slope = 0.64 (Lower 95%: 0.58, Upper 95%: 0.68).

**Figure 3** demonstrates the scatterplot of the length and width measurements for 250 seeds obtained by microscope and a Samsung camera using daylight lamps (L3) and sunlight (L6) lighting conditions. This figure demonstrates that with good lighting conditions, the grain size estimates obtained by the mobile device are in agreement with the microscope measurements. However, in sunlight conditions, our software tends to underestimate the grain dimensions for larger grains and overestimate them for smaller grains. This effect is likely due to a shadow effect that introduces systematic bias in the grain size estimation when an image is taken under direct bright sunlight.

We estimated the time used for the analysis of a single image by mobile devices and SmartGrain software at different image resolutions. The results are shown in Supplementary Table S3. The time for low resolution image processing (2592 × 1944 pixels) is approximately 30 s. For a higher resolution camera (Samsung 3264 × 2448), this value is close to 1 min. Interestingly, this is comparable with the time of image processing by SmartGrain (at similar resolutions, 3510 × 2550).

**FIGURE 4:**
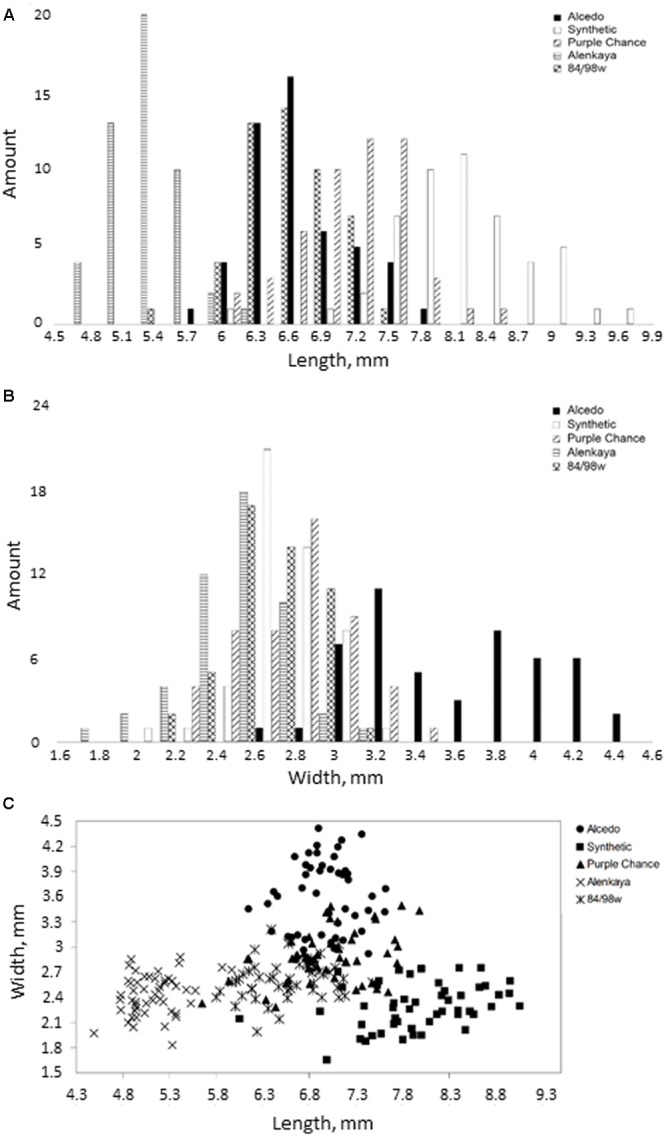
**Distribution of grain width and length for five different wheat varieties. (A,B)** The histograms of the length and width distribution, respectively; **(C)** 2D-scatter plot of grains from different varieties in length (*X*-axis) and width (*Y*-axis).

Using the SeedCounter mobile application, we performed wheat grain morphometry of five varieties. For each variety, 50 grains were analyzed, and their length and width were measured. The results are shown in **Figures 4A–C**.

The diagrams in **Figures 4A–C** demonstrate the reliability of discriminating grains from different wheat varieties based on their length and width estimates. The figure shows that the Alcedo cultivar has the thickest grains (average width–3.59 mm) and that the Synthetic cultivar has the longest grains (7.97 mm). The separation of varieties by seed size is clearly demonstrated in **Figure 4C**, where different varieties occupy different plot areas.

## Discussion

Image processing methods for seed morphometry and classification have been implemented since the 1980s (Sapirstein et al., 1987). Updates of these methods appear constantly, including in recent years (Smykalova et al., 2013; Whan et al., 2014; Miller et al., 2016; Sankaran et al., 2016). New methods use various optical sensing techniques to estimate seed quality and safety (Huang et al., 2015), describe complex seed shapes using 2D images (Williams et al., 2013; Cervantes et al., 2016). Breakthrough 3D imaging technology and robotics (Jahnke et al., 2016; Roussel et al., 2016) or X-ray computed tomography (Strange et al., 2015) implemented for evaluating seed shape in fine detail. However, there is still a need for seed phenotyping using simple and low cost tools (Whan et al., 2014). They can be effectively implemented with high throughput. Despite simplicity, they are powerful enough to identify QTL related to seed morphology and size (Gegas et al., 2010; Herridge et al., 2011; Moore et al., 2013; Williams et al., 2013). Mobile devices are valuable tools in this regard. They provide the researcher everything needed for simple phenotyping, including a digital camera, a powerful processor, and Internet access. They can be applied far from the lab, yet provide reasonable precision for phenotypic parameter estimates. Mobile devices are also convenient for the novel type of plant phenotyping ‘by crowd’ (Rahman et al., 2015).

We suggest a program for grain morphometry using mobile devices. The protocol of the analysis setup is simple and uses a white paper sheet of standard size as a background to convert pixels into the metric scale. To test the accuracy of the program, we performed a series of image analysis experiments using three types of mobile devices and six lighting conditions. In our work, the mean absolute errors of the length/width estimates are approximately 7–10% and correlation coefficients for length and width between estimated and actual values at ambient lighting are close to 0.93 and 0.77, respectively. Similar analysis performed in a recent work, Miller et al. (2016) reported *r*^2^ = 0.996 for maize kernel length estimated from digital images and their actual values (flatbed document scanner Epson V700, 1200 dpi image, 24-bit color resolution). Sankaran et al. (2016) reported Pearson correlation coefficients between image-based estimates of chickpea seed size and their real values ranging from 0.86 to 0.93 (Canon 70D digital SLR camera, tripod setup, 15–85 mm zoom lens, image resolution set to 2700 × 1800 pixels). Whan et al. (2014) analyzed performance of wheat seed length and width measurements by the following three methods: GrainScan (developed by the authors), SmartGrain (Tanabata et al., 2012), and SeedCount (Next Instruments, 2015). They used an Epson Perfection V330 (Seiko Epson Corporation, Suwa, Japan) scanner to obtain 300 dpi color images. Whan et al. (2014) demonstrated that the average accuracy (Pearson correlation between true parameters and image-based estimates) for GrainScan was very high (0.981–0.996), while the average accuracy for SmartGrain was lower (0.871–0.947), similarly to that of SeedCount at the ambient light conditions (0.731–0.940; Supplementary Table S2). Note, the accuracy for length estimates was higher than for width for all three methods. Our results demonstrate that SeedCounter accuracy and efficiency are comparable with those obtained using desktop PC/scanner/camera devices. Note that we used cameras with moderate resolution and unpretentious lighting conditions for our experiments.

Interference from uncontrolled or uneven lighting is the most basic challenge for smartphone optical sensing (McCracken and Yoon, 2016). We found that the lighting of the paper has significant influence on the accuracy of our method, unlike the smartphone type (**Table 4**; Supplementary Table S2). We used ANOVA with six different classes of lighting not related directly to luminosity. We chose this approach because our data demonstrated that the influence of luminosity itself on accuracy is not straightforward: images taken at high luminosity under direct sunlight demonstrate increased error in comparison with medium luminosity images and ambient lighting. Under low light conditions (11-W daylight lamp or without artificial lighting), grain number estimation accuracy decreases. Lighting conditions with halogen and daylight lamps (experimental conditions of Sam_L4, Sony_L4, and DNS_L4) caused a small shimmering effect on the images. This effect can complicate the paper recognition process and lead to distorted results. The flicker effect was also present under Sam_L3 and DNS_L3 conditions but could be significantly suppressed using the “night shot” technique. The location of light sources and their angle with the paper surface can distort the measurements and degrade sheet recognition conditions. A brighter, diffused light eliminates distortions associated with the appearance of dark spots on the borders of the sheet that can be incorrectly recognized as grains, allows for more efficient separation of touching grains and reduces the likelihood that the grain in the image will merge with the background.

There are several approaches suggested to improve image quality and analysis precision. Some of them require auxiliary/add-on devices (enclosed lighting and imaging attachments) to improve the sensitivity of the smartphone camera (Barbosa et al., 2015). Other methods implement normalization algorithms to reduce lighting inhomogeneity on the image (McCracken et al., 2016). There is still no perfect solution to this problem and further investigation is required to reduce image processing errors from these sources (McCracken and Yoon, 2016).

Mobile applications can significantly accelerate the process of counting the number of grains of wheat in an ear. The time required to calculate approximately 50 grains using a mobile device is approximately 20–55 s, depending on the mobile device and camera resolution. The time required for manually counting the same number of grains may be a little less but mobile devices allow processing a series of images in the background and automatically saving and transmitting data to the server. Increasing the number of grains to 100 increases the running time of the algorithm by 5–10 s. The time required to evaluate the lengths and widths of 50 grains under the microscope is approximately 40–60 min. The mobile application performs this analysis in approximately 1 min.

Thus, the mobile application “SeedCounter” allows for the large-scale measurement of the phenotypic parameters of wheat grains, such as length, width, area, and number of grains per ear, both in “the field” and in the laboratory.

## Author Contributions

EK developed algorithms, SeedCounter software, and performed data analysis. MG contributed to algorithm development and data analysis. DA conceived of the study and participated in its design. All authors participated in writing the manuscript as well as read and approved its final version.

## Conflict of Interest Statement

The authors declare that the research was conducted in the absence of any commercial or financial relationships that could be construed as a potential conflict of interest.

## Footnotes

1 https://play.google.com/store/apps/details?id=org.wheatdb.seedcounter
