# Supplementary material for: Evaluation of the SeedCounter, A Mobile Application for Grain Phenotyping
Source: Front Plant Sci. 2017 Jan 4;7:1990. doi: 10.3389/fpls.2016.01990 (PMC5209368; doi:10.3389/fpls.2016.01990)
Supplement: Supplementary file 1 [file Table_1.docx]

**Table S1.** Characteristics of mobile devices used and their camera resolutions.

| Mobile device | Operating system | Processor (core x frequency) | RAM | Camera resolution |
| --- | --- | --- | --- | --- |
| Sony Ericsson  Experia pro mini | Android 2.3 | Qualcomm MSM 8255  (1×1000 MHz) | 512MB | 5 Mpx  (2592×1944) |
| DNS AirTab  m101w | Android 4.1 | RockChip RK3066  (2×1500 MHz) | 1GB | 5 Mpx  (2592×1944) |
| Samsung Galaxy  Grand 2 | Android 4.3 | ARM Cortex-A7  (4×1200 MHz) | 1.5GB | 8 Mpx (3264×2448) |
