# Supplementary material for: Evaluation of the SeedCounter, A Mobile Application for Grain Phenotyping
Source: Front Plant Sci. 2017 Jan 4;7:1990. doi: 10.3389/fpls.2016.01990 (PMC5209368; doi:10.3389/fpls.2016.01990)
Supplement: Supplementary file 2 [file Table_2.docx]

**Table S2.**  The average accuracy values of the width and length estimates by SeedCounter mobile application under different lighting conditions.

| Lighting condition | Average MAE (mm) | Average MAPE | Average *r_l_* | Average *r_w_* |
| --- | --- | --- | --- | --- |
| L1 | 0,297 | 7,861 | 0,937 | 0,785 |
| L2 | 0,301 | 7,795 | 0,931 | 0,790 |
| L3 | 0,289 | 7,619 | 0,940 | 0,793 |
| L4 | 0,322 | 8,318 | 0,928 | 0,782 |
| L5 | 0,350 | 8,535 | 0,867 | 0,731 |
| L6 | 0,344 | 8,416 | 0,888 | 0,762 |
