# Supplementary material for: Evaluation of the SeedCounter, A Mobile Application for Grain Phenotyping
Source: Front Plant Sci. 2017 Jan 4;7:1990. doi: 10.3389/fpls.2016.01990 (PMC5209368; doi:10.3389/fpls.2016.01990)
Supplement: Supplementary file 3 [file Table_3.DOCX]

**Table S3.** Time used for single image processing for different mobile devices and SmartGrain software.

| Application | Device | Resolution (pixels) | Time (seconds) |
| --- | --- | --- | --- |
| SeedCounter | Samsung | 3264×2448 | 55 |
| SeedCounter | Sony | 2592×1944 | 30 |
| SeedCounter | DNS | 2592×1944 | 25 |
| SmartGrain | PC | 3510×2550 (300 dpi) | 55 |
| SmartGrain | PC | 5100×7020 (600 dpi) | 220 |
